# Description and Predictive Factors of Individual Outcomes in a Refugee Camp Based Mental Health Intervention (Beirut, Lebanon)

**DOI:** 10.1371/journal.pone.0054107

**Published:** 2013-01-17

**Authors:** Pierre Bastin, Mathieu Bastard, Ludovic Rossel, Pablo Melgar, Alison Jones, Annick Antierens

**Affiliations:** 1 Médecins sans Frontières, Geneva, Switzerland; 2 Epicentre, Paris, France; The University of Hong Kong, Hong Kong

## Abstract

**Background:**

There is little evidence on the effectiveness of services for the care of people with mental disorders among refugee populations. Médecins sans Frontières (MSF) has established a mental health centre in a mixed urban-refugee population in Beirut to respond to the significant burden of mental health problems. Patients received comprehensive care through a multidisciplinary team. A cohort of people with common and severe mental disorders has been analysed between December 2008 and June 2011 to evaluate individual outcomes of treatment in terms of functionality.

**Methods:**

All patients diagnosed with mental disorders were included in the study. The Global Assessment of Functioning (GAF) and the Self Reporting Questionnaire–20 items (SRQ 20) were used as tools for baseline assessment, monitoring and evaluation of patients. Predictors of evolution of SRQ20 and GAF over visits were explored using a linear mixed model.

**Results:**

Up to June 2011, 1144 patients were followed, 63.7% of them Lebanese, 31.8% Palestinians and 1.2% Iraqis. Females represented 64.2% of the patient population. Mean age was 39.2 years (28.5–46.5). The most frequent primary diagnoses were depressive disorders (28.8%), anxiety disorders (15.6%) and psychosis (11.5%). A lower baseline SRQ20 score/higher baseline GAF score (indicators of severity), being diagnosed with anxiety (compared to being diagnosed with depression or psychosis) and a higher level of education were associated with better outcomes.

**Discussion:**

In this MSF program, we observed a significant decrease of SRQ20 individual scores and a significant increase of individual GAF scores. This corresponded to an improvement in the functionality of our patients. Analysis of the predictors of this positive evolution indicates that we need to adapt our model for the more severe and less educated patients. It also makes us reflect on the length of the individual follow-up. Further research could include a qualitative evaluation of the intervention. Results of this study have been presented at the World Congress of the World Federation for Mental Health in Cape Town, October 2011.

## Introduction

An estimated 450 million people worldwide have a mental disorder, 85% of them living in low and middle income countries. At any given time, approximately 10% of adults are experiencing a current mental disorder, and 25% will develop one at some point during their lifetime [1], [2]. Effective treatments are available for a range of mental disorders; medication and psychological interventions. In most countries, especially those with low- and middle-income economies, there is an enormous gap between those who need mental health care, on one hand, and those who receive care, on the other hand. The causes of mental health disorders are widespread and understandable. Difficult socio-economic conditions are associated with significantly higher levels of mental health problems and mental illness [3], especially in protracted refugee situations [4], [5].

A middle income country with a population of 4.35 million, Lebanon has experienced multiple wars, internal conflicts, and a long period of instability. Internal and external displacement has occurred through the years and, today, among the refugee population, around 400 000 are Palestinians. The country has a heavily privatized and fragmented health system. Uncovered medical needs are found in mental health [6], geriatric care, and non-communicable diseases. It is known as a hot zone for illicit drug production and use. The context has the characteristics of middle income countries; access to technicity (or technology), but with important inequalities. In terms of prevalence of mental disorders, a national epidemiological psychiatric survey in the Lebanese population had shown that 17% (SE: 1.6) of those interviewed met criteria for at least one DSMIV/CIDI disorder [7]. For the Palestinian population, a prevalence study made in the camp of Burj-el-Barajneh during the summer of 2010, revealed 29% (CI: 19%–39%) diagnosed with a mental disorder. The study also showed important levels of associated disability, enormous treatment gaps and unavailability of mental health services (unpublished data).

Médecins sans Frontières Switzerland (MSF) has established a community mental health centre in Burj-el-Barajneh, working in close collaboration with Primary Health Care (PHC) services for Palestinian and Lebanese populations [8]. The centre was opened on December 18, 2008, aiming at a progressive integration of the mental health activities inside United Nations Relief and Works Agency (UNRWA) health services and a Palestinian Red Crescent Society (PRCS) hospital.

There is little evidence on the effectiveness of services for care of people with common and severe mental disorders among refugee populations [9]. The objective of this study was to describe the individual outcomes of patients in terms of functionality and to identify potential predictors of these outcomes.

## Methods

### Study Design

The MSF project welcomes any adult (>18 years) in the community regardless of nationality, religion or beliefs. Each patient coming for treatment to the center was asked to answer the questionnaires included in the patient file.

In Burj-el-Barajneh, a suburban area in South West Beirut, there are Palestinians from the camp as well as Lebanese and other refugees (Iraqi). We estimate a general population of 20 000 people living in the camp and 200 000 people around the camp (Burj-el-Barajneh area).The centre is located just outside the camp.

Patients came to the center either by their own initiative or were referred by family or friends (thanks to our Community Health Workers network), another organisation, the UNRWA clinic, their general practitioner or our social workers (SW). On arrival, patient was first seen by a MSF psychiatric nurse who evaluated the indication and decided whether the patient should go to the psychologist first or directly to the psychiatrist. The nurse also checked the general health status. MSF psychologist then decided on the setting needed: individual, couple, family, group or art therapy. Finally, MSF psychiatrist saw the patient, if necessary, for confirmation of diagnosis and provision of psychotropic medication. All the diagnosis were done following the International Classification of Diseases (ICD-10).Some speech and self-help groups as well as relaxation sessions were also organised in the centre, animated by MSF SW or Community health workers (CHW). When indicated, a SW assessment and intervention was performed (home visits). Patients were followed-up every 2 weeks (sometimes weekly) by the psychologist and every month by the psychiatrist. If needed, patients could also come to the center for emergency.

All patients coming to the centre, UNRWA clinic and PRCS hospital between December 2008 and June 2011 were included in the analysis.

### Outcome Measurements

At first visit and during follow-up consultations, two functionality questionnaires were passed. The first one was the Self Reporting Questionnaire–20 items (SRQ 20), based on patient’s statement. This instrument was designed by the World Health Organization to screen for psychiatric disturbance in primary health care settings, especially in developing countries [10]. The score ranges from 0 to 20, scoring 1 for a positive answer and 0 for a negative answer. Examples of questions are: “Do you feel nervous, tense or worried?”, “Do you find it difficult to enjoy your daily activities?”, or “Has the thought of ending your life been on your mind?” The second questionnaire was the Global Assessment of Functioning (GAF) [11], based on clinical evaluation. This instrument considers the client’s psychological, social, and occupational functioning on a hypothetical continuum (1–100) of mental health-illness, in intervals of 10. For example, the interval 61–70 means: “Some mild symptoms (e.g., depressed mood and mild insomnia) OR some difficulty in social, occupational, or school functioning (e.g. occasional truancy, or theft within the household), but generally functioning pretty well, has some meaningful interpersonal relationships”. The GAF was routinely evaluated by the psychologist at each consultation and the SRQ20 was performed monthly. Both scales have been translated in Arabic and translated back in English.

### Statistical Analysis

Closing date of the database was June 30, 2011. Baseline characteristics of the patients were recorded for all the patients included in the study and for specific subgroups of the cohort. Categorical variables were summarized using counts and percentages, and continuous variables were summarized using median and interquartile range (IQR).

We estimated the median evolution of SRQ20 and GAF over time. Differences between scores at first visit and at last visit were reported and tested using Wilcoxon rank sum test. We explored potential predictors of SRQ20 and GAF scores using random-linear mixed models. Factors included in univariate analysis were age, gender, nationality, level of education, employment and marital status, having access to several services (electricity, water, heating), score at first consultation, diagnosis at first consultation, medical coverage, personal and financial support, and period of inclusion in the programme. Multivariate analysis was then performed using a backward approach.

All analyses were performed with Stata 10 software (Stata Corporation, College Station, TX). The threshold P-value to include factors in an initial model was 0.4 and 0.05 for all other tests.

### Ethical Review

This retrospective analysis was performed from an anonymized database. All patients gave their oral consent before answering the questionnaires which had been previously sent for approval to the local political and religious authorities. The World Medical Association’s Declaration of Helsinki was respected. This study has met the Medecins Sans Frontieres’ Ethics Review Board-approved criteria for analysis of routinely-collected program data.

## Results

A total of 1144 patients were included in the study. Baseline characteristics of the patients are presented in Table 1. Among the 1144 patients, 729 (63.7%) were Lebanese, 364 (31.8%) were Palestinians and 14 (1.2%) were Iraqi refugees. The proportion of Palestinians included in our program increased from 26.5% for the period December 2008 – May 2009 to 33.3% for the period December 2010 – June 2011. Among the 1144 patients, 734 were women (64.2%). The proportion of males included in our program increased from 34.4% for the period December 2008 – May 2009 to 41.7% for the period June 2010 – November 2010.The median age of all patients was 39 years [IQR 28.5–46.5]. The distribution of inclusions in the programme was as follows: 151 patients have been included for the period December 2008 – May 2009, 173 for the period June 2009 – November 2009, 255 for the period December 2009 – May 2010, 180 for the period June 2010 – November 2010, and 385 for the period December 2010 – June 2011. About 57.3% of the patients were under 40 while 32.6% belonged to the age category 41–60. Looking at the references: 46% were referred to the centre by family or neighbours, 12% by another organization, 10.4% by our direct partner UNRWA, 9.8% from home visits and 6.1% by their medical doctor. In terms of primary diagnosis, 28.8% of the patients were identified as suffering from depression, 16% from anxiety and 12% from psychosis. In Table 1, we also displayed baseline characteristics of the patients included in the analysis of SRQ20 and GAF evolution. A total of 526 patients (46%) had at least 2 measures of GAF and 38.5% had at least 2 measures for SRQ 20.There is no evidence for strong differences in baseline characteristics between patients included in the different analyses and the overall cohort.

**Table 1:** Baseline characteristics of patients included in the Burj-el Barajneh cohort between December 2008 and June 2011.

Characteristics	Cohort (N = 1144)	At least two GAFmeasurements (N = 526)	At least two SRQ20 measurements (N = 441)
**Nationality, n (%)**			
Lebanese	729 (63.7)	399 (75.8)	331 (75.0)
Palestinian	364 (31.8)	113 (21.5)	97 (22.0)
Iraqi	14 (1.2)	4 (0.8)	3 (0.7)
Other	32 (2.8)	10 (1.9)	10 (2.3)
Missing	5 (0.4)	0 (0.0)	0 (0.0)
**Gender, n (%)**			
Men	410 (35.8)	192 (36.5)	126 (28.6)
Women	734 (64.2)	334 (63.5)	315 (71.4)
**Age (years), n (%)**			
Median [IQR]	39.2 [28.5–46.5]	39.6 [30.5–48.2]	40.0 [30.6–48.4]
18–40	655 (57.3)	273 (51.9)	223 (50.6)
41–60	373 (32.6)	201 (38.2)	173 (39.2)
>60	97 (8.5)	44 (8.4)	38 (8.6)
Missing	19 (1.6)	8 (1.5)	7 (1.6)
**Reference, n (%)**			
Family	526 (46.0)	272 (51.7)	218 (49.4)
NGO	137 (12.0)	58 (11.0)	51 (11.6)
UNRWA	119 (10.4)	48 (9.1)	43 (9.8)
Home visit	112 (9.8)	21 (4.0)	18 (4.1)
GP	70 (6.1)	44 (8.4)	36 (8.2)
Other	120 (10.5)	61 (11.6)	56 (12.6)
Missing	60 (5.2)	22 (4.2)	19 (4.3)
**Diagnosis, n (%)**			
Depression	329 (28.8)	198 (37.6)	207 (46.9)
Anxiety	178 (15.6)	114 (21.7)	117 (26.5)
Psychosis	132 (11.5)	91 (17.3)	21 (4.8)
Other	215 (18.8)	121 (23.0)	93 (21.1)
Missing	290 (25.3)	2 (0.4)	3 (0.7)

**Figure 1:**
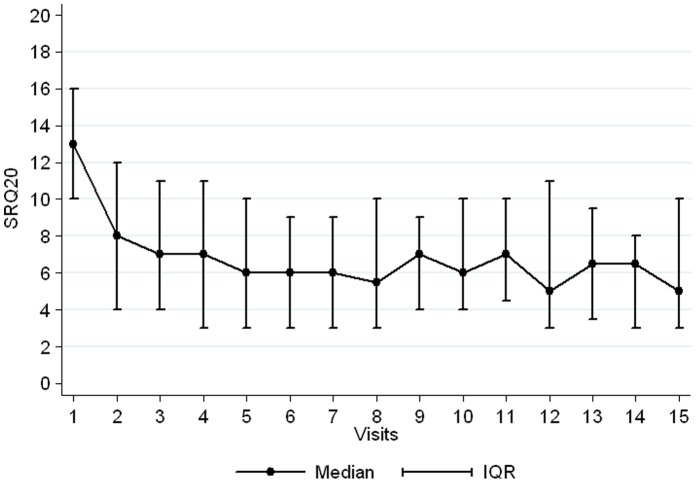
Evolution of SRQ20 score over visits to the centre for patients included in the Burj-el Barajneh cohort between December 2008 and June 2011.

Patients with at least 2 measures for SRQ20 were followed up for a median of 8 months (IQR 3–16) and attended a median of 4 visits (IQR 2–7). The median SRQ20 score at baseline was 13 [IQR, 10–16]. A drastic decrease was observed directly after the first visit and the SQR20 tended to stabilize at a median of 6 after the fifth visit (Figure 1). The median SRQ20 score at last visit for each patient was 7 [IQR 4–12] and was significantly lower than at first visit (Wilcoxon test p<0.001).

We presented in Table 2 the factors associated with the evolution of SRQ20 over time. A higher baseline score was associated with a higher score over time while an older age was associated with a lower score over time. Compared to patients diagnosed with depression, those diagnosed with anxiety had a lower SRQ20 score over time. Finally, a higher level of education was also associated with a lower SRQ20 score over time.

**Table 2:** Predictors of the evolution of SRQ20 scores over time for patients included in the Burj-el Barajneh cohort between December 2008 and June 2011.

Predictors	Adjusted coefficient* (95% CI)
**Baseline SRQ20 score (1 unit increases)**	0.33 (0.26; 0.41)
**Age per 10 unit increases (years)**	−0.46 (−0.72; − 0.20)
**Diagnosis**	
Depression	Reference
Anxiety	−0.99 (−1.75; −0.23)
Personality disorder	−0.15 (−1.33; 1.02)
Psychosis	−0.69 (−2.27; 0.90)
Bipolar disorder	−0.86 (−2.20; 0.48)
Other	
**Level of education**	
Illiterate	Reference
Primary	−1.85 (−3.15; −0.54)
University	−2.86 (−4.22; −1.49)

* adjusted for gender, time of follow-up and period of inclusion.

**Figure 2:**
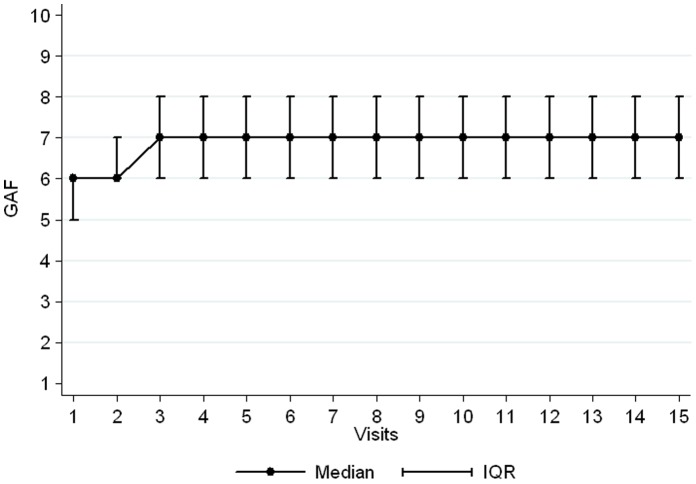
Evolution of GAF score over visits to the centre for patients included in the Burj-el Barajneh cohort between December 2008 and June 2011.

Patients with at least 2 measures for GAF were followed up for a median of 11 months (IQR 3–18) and attended a median of 5 visits (IQR 3–9). The median GAF score at baseline was 6 [IQR, 5–6]. A small increase was observed during second and third visit before stabilizing at a median of 7 (Figure 2). The median GAF score at last visit for each patient was 6 [IQR 6–8] and was significantly higher than at first visit (Wilcoxon test p<0.001). We have used a non parametric test (the Wilcoxon rank sum test) to compare the distribution of the GAF between baseline score and score at last visit. This means that at baseline, only 25% of patients had a GAF score ≥6, while at last visit, 75% of patients had a GAF score ≥6. We presented in Table 3 the factors associated with the evolution of GAF over time. Having a baseline GAF score ≥5, being diagnosed with anxiety compared to depression, and having a higher level of education was significantly associated with a higher GAF score over time. Patients diagnosed with psychosis had a lower score GAF over time than those diagnosed with depression.

**Table 3:** Predictors of the evolution of GAF scores over time for patients included in the Burj-el Barajneh cohort between December 2008 and June 2011.

Predictors	Adjusted coefficient*(95% CI)
**Baseline GAF score**	
<5	Reference
≥5	0.54 (0.36; 0.72)
**Diagnosis**	
Depression	Reference
Anxiety	0.25 (0.01; 0.48)
Personality disorder	0.00 (−0.37; 0.38)
Psychosis	−0.49 (−0.76; −0.22)
Bipolar disorder	0.14 (−0.24; 0.42)
Other	
**Level of education**	
Illiterate	Reference
Primary	0.50 (0.15; 0.85)
University	0.71 (0.34; 1.08)

* adjusted for age, gender, time of follow-up and period of inclusion.

## Discussion

In this MSF program, we observed a decrease of SRQ20 individual scores and an increase of individual GAF scores. This corresponded to an improvement in the functionality of our patients, indicating the success of treatments.

Treating mental disorders as early as possible, holistically and close to person’s home and community leads to the best health outcomes [12]. Primary care offers unparalled opportunities for prevention of mental disorders and mental health promotion, for family and community education, and for collaboration with other sectors [12], [13].

We observed a progressive increase of inclusion over time, proportionate to the acceptance and information about the centre, thanks to “word of mouth” as well as the services’ promotion made by our CHWs. Collaboration with other organizations (including UNRWA) becomes relatively effective in referring patients to the center and the camp’s clinics. The work performed by the CHWs and SWs inside the patients’ homes also brought patients to our services. Referrals from external doctors remained comparatively low.

We observed a progressive increase in the proportion of males coming to our services, as contacts with the religious authorities were reinforced.

Most of our patients were Lebanese, which is not surprising given the centre’s location and the density of population in Burj-el-Barajneh. We estimate that, even if a prevalence study were not performed there, the needs and gaps regarding mental health services would be almost identical (deprived area, with a lot of displaced population) to those found inside the camp.

The proportion of Palestinians coming to the services has risen while those services were progressively integrated in the camp health structures.

Evolutions of both scales can be considered as satisfying. As the cut-off score for SRQ 20 was arbitrarily set at 7 (based on WHO document [11] and other field observations), we could consider our intervention brought most of the patients under this line. Stabilization around 6 is, in this sense, a very good result.

After several visits we no longer observed an effect. This could be the stabilization phase, corresponding to the maintenance treatment of chronic patients, but this could also questions the necessity to perform more than 6 to 8 consultations.

Being unable to test for all possible predictors, we used other studies as a base to examine which predictors to explore [14]. Our intervention appears better adapted to patients suffering from anxiety than to those with severe disorders like psychosis. The baseline score is also a predictor of evolution over time tending to show we do better with less severe cases. A big and progressive effect was observed for the educational level as a predictor of a favourable evolution. This leads us to think we should increase our attention and adapt our interventions to severe mental disorders (SMD) and less educated patients.

Patient file which included both scales was tested beforehand on healthy volunteers. Those scales have been chosen in agreement with the clinicians of the project. We are considering keeping only one of the instruments for monitoring of future projects. We would prefer to opt for SRQ even if it is not adapted to severe (psychotic) patients, nor always supported to follow patients’ evolution (as a monitoring tool). The narrow distribution of GAF (between intervals 50 to 80) and the inter-rater problems of reliability create more limitations in the use of GAF only. Another problem faced here with the GAF is that we do not observe any further changes after 3 visits.

Some colleagues from other MSF sections use 1–10 Likert scales, complaint and functionality rating (patient and clinician point of view), which are very practical, but with the disadvantage of being not yet standardized tools [14].

For common mental disorders (CMD) there would be other possibilities such as the depression and anxiety stress scale (DASS) while for psychosis we could consider using an adaption of the positive and negative syndrome scale (PANSS) or the brief psychiatric rating scale (BPRS). Actually we may have to use different instruments for CMD and SMD.

Our study presents several limitations. First, a moderate proportion of patients have comparative measures (38.5% for SRQ20 and 46% for GAF). As in each cohort, we probably have a selection bias. The patients remaining in the programme (and for whom we have comparative measures) are the ones doing better.

We have tried to collect a lot of information through the patient file on top of SRQ 20 and GAF (a questionnaire on health behavior, the Self Functioning questionnaire with 12 items, the Harvard Trauma Scale, and the Trauma Scale Questionnaire). It is certainly one of the reasons why the questionnaires were not always filled. From this experience we believe that the project team’s efforts in collecting information can be sustained for a limited period of time (pilot or research project) but that this information should be limited to the minimum needed for effective monitoring of a regular project and should also be timely analyzed at field level.

### Conclusion

The aim of this study was to describe individual outcomes of patients and to identify potential predictors of these outcomes in a refugee camp based mental health intervention. We believe that the efficacy of our treatment model has been demonstrated by the positive individual outcomes we got in terms of functionality. Analysis of predictors of this positive evolution shows that we need to adapt our model for the more severe and less educated patients. It also makes us reflect on the length of the individual follow-up. Those results must be taken into consideration in our other interventions, with limitations due to the context. This study provides evidence that SRQ20 can be used as a baseline and monitoring tool but that the use of GAF for the same purpose is questionable.

A further and deeper analysis of the data collected would be needed, especially regarding the link between socio-economic determinants and patients’ outcomes. We do indeed consider social determinants of mental health as predictors of mental health conditions (in protracted refugee situations in general). Advocating for better socio-economic conditions for the refugee populations is also part of our work.

Further research could also include a qualitative evaluation of the intervention.
